# A Case Series Clinical Trial of a Novel Approach Using Augmented Reality That Inspires Self-body Cognition in Patients With Stroke: Effects on Motor Function and Resting-State Brain Functional Connectivity

**DOI:** 10.3389/fnsys.2019.00076

**Published:** 2019-12-17

**Authors:** Fuminari Kaneko, Keiichiro Shindo, Masaki Yoneta, Megumi Okawada, Kazuto Akaboshi, Meigen Liu

**Affiliations:** ^1^Department of Rehabilitation Medicine, Keio University School of Medicine, Tokyo, Japan; ^2^Department of Rehabilitation, Shonan Keiiku Hospital, Fujisawa, Japan; ^3^Hokuto Social Medical Corporation, Obihiro, Japan

**Keywords:** stroke, rehabilitation, kinesthetic illusion, virtual reality, body ownership, embodied visual feedback, resting-state brain functional connectivity, mirror visual feedback

## Abstract

Barring a few studies, there are not enough established treatments to improve upper limb motor function in patients with severe impairments due to chronic stroke. This study aimed to clarify the effect of the kinesthetic perceptional illusion induced by visual stimulation (KINVIS) on upper limb motor function and the relationship between motor function and resting-state brain networks. Eleven patients with severe paralysis of upper limb motor function in the chronic phase (seven men and four women; age: 54.7 ± 10.8 years; 44.0 ± 29.0 months post-stroke) participated in the study. Patients underwent an intervention consisting of therapy using KINVIS and conventional therapeutic exercise (TherEX) for 10 days. Our originally developed KiNvis™ system was applied to induce KINVIS while watching the movement of the artificial hand. Clinical outcomes were examined to evaluate motor functions and resting-state brain functional connectivity (rsFC) by analyzing blood-oxygen-level-dependent (BOLD) signals measured using functional magnetic resonance imaging (fMRI). The outcomes of motor function (Fugle-Meyer Assessment, FMA) and spasticity (Modified Ashworth Scale, MAS) significantly improved after the intervention. The improvement in MAS scores for the fingers and the wrist flexors reached a minimum of clinically important differences. Before the intervention, strong and significant negative correlations between the motor functions and rsFC of the inferior parietal lobule (IPL) and premotor cortex (PMd) in the unaffected hemisphere was demonstrated. These strong correlations were disappeared after the intervention. A negative and strong correlation between the motor function and rsFC of the bilateral inferior parietal sulcus (IPS) significantly changed to strong and positive correlation after the intervention. These results may suggest that the combination approach of KINVIS therapy and TherEX improved motor functions and decreased spasticity in the paralyzed upper extremity after stroke in the chronic phase, possibly indicating the contribution of embodied-visual stimulation. The rsFC for the interhemispheric IPS and intrahemispheric IPL and PMd may be a possible regulatory factor for improving motor function and spasticity.

**Clinical Trial Registration**: www.ClinicalTrials.gov, identifier NCT01274117.

## Introduction

Upper extremity motor function after stroke recovers in only 50% of all survivors at 6 months post-stroke (Kwakkel et al., 2003). Therefore, maximizing recovery of motor function in the upper extremity after stroke is socially meaningful (Langhorne and Legg, 2003), but is a challenge for the area of rehabilitation science. Constraint-induced movement therapy (CIMT) involves ipsilesional limb restraint with the training of the paretic arm (Wolf et al., 2006). There is moderate evidence of the therapeutic effects of CIMT; however, this therapy is adaptive for patients with more than moderate paralysis and not for patients with severe paralysis. In addition, mirror therapy (Dohle et al., 2009), mental practice (Page et al., 2007), and virtual reality therapy (Klamroth-Marganska et al., 2014) are shown to have beneficial effects with moderate-quality evidence (Pollock et al., 2014). All these therapies have been implemented in patients with dexterity (Kwakkel et al., 2003; Langhorne and Legg, 2003; Wolf et al., 2006; Page et al., 2007; Dohle et al., 2009; Klamroth-Marganska et al., 2014; Pollock et al., 2014). Recently, clinical trials for patients with stroke in the chronic phase were reported. The effect size of the mirror therapy (Colomer et al., 2016) or functional electrical stimulation (Carda et al., 2017) intervention may be insufficient. Additionally, clinical trials using the brain-machine interface have demonstrated a higher effect size (Ramos-Murguialday et al., 2013, 2019). Although a few influential results have been reported, other significant therapeutic approaches for patients with chronic stroke should be developed.

Nerve fiber tracking, which is performed using diffusion tensor tractography, is often used clinically and demonstrates the absence of fibers in the tract descending from the primary motor (M1) and somatosensory (S1) cortices to the brain stem. Correlation analyses between infarct volume (Saver et al., 1999) and location with motor impairment indicate that damage in the motor areas, corona radiata, and internal capsule decrease the probability of upper limb functional recovery (Binkofski et al., 1996; Shelton and Reding, 2001; Crafton et al., 2003; Schiemanck et al., 2008; Johansen-Berg et al., 2010; Moulton et al., 2015). Several functional magnetic resonance imaging (fMRI) studies have analyzed blood-oxygen-level-dependent (BOLD) signals as a measure of resting-state brain functional connectivity (rsFC) among brain sites (Carter et al., 2010; Urbin et al., 2014). However, these studies have not discussed the classification of the time course of recovery from a stroke or the recovery stages of sensory-motor function. In the acute phase after stroke, the inter- and intra-hemispheric rsFC differ depending on the severity of the stroke and become weak in patients with severe conditions (De Bruyn et al., 2018). In patients in the subacute-to-chronic phase after stroke, the rsFC between M1 of each hemisphere decreases compared with that in healthy control individuals (Zhang et al., 2016). In a previous study, the rsFC between S1 and other regions indicated an asymmetrical difference, and the index of asymmetry significantly correlated with motor function deficits (Frías et al., 2018).

We have conducted clinical studies including patients with severe motor paralysis to induce recovery of motor function using kinesthetic illusion induced by visual stimulation (KINVIS). KINVIS is defined as the psychological phenomenon in which a person who is resting feels as if a part of his/her own body is moving or feels the desire to move a body part while watching a movie of the body part that is moving. To establish the therapeutic utility of this phenomenon, we have developed a system that is clinically useful, named KiNvis™. In KINVIS therapy, the patient experiences KINVIS while neuromuscular electrical stimulation is applied to the muscle that is an agonist to the one whose movement is being watched in the movie. Therefore, KINVIS therapy indicates a combination of KINVIS with neuromuscular electrical stimulation. This system can be viewed as an intervention of virtual reality (augmented reality) using embodied-visual feedback, which can induce embodied cognitive change in self-body and physiological effects on motor-associated areas in the brain (Kaneko et al., 2007, 2015, 2016a,b; Aoyama et al., 2012). In other words, this may represent a cognitive stimulation to the embodied brain system for body ownership, a sense of agency, and kinesthetic perception. A specific brain network is activated during KINVIS (Kaneko et al., 2015). The first report of the physiological effects of embodied-visual feed-back indicated an enhancement of M1 excitability (Kaneko et al., 2007), which is sustained after KINVIS therapy (Kaneko et al., 2016a). Frequently, while the subject is experiencing KINVIS, a spontaneous muscular contraction can be observed in the agonist muscle that is shown in the movie (Itaguchi, 2018, unpublished data). We consider that these previous studies suggest that KINVIS may be associated with the drive of the nervous system for motor output. Moreover, a previous feasibility study showed that motor function immediately changes in patients with stroke exhibiting severe paralysis (Kaneko et al., 2016b). Therefore, we speculated that the repetitive application of KINVIS therapy in addition to conventional therapeutic exercise (TherEx) could produce a positive effect on motor function, with enhanced brain plasticity, in patients with stroke. We hypothesized that the connectivity of brain regions of interest (ROIs) that are associated with sensory-motor function and KINVIS might improve and induce changes in motor function.

Thus, the purpose of this study was to clarify the effect of repetitive KINVIS therapy in combination with TherEx on upper limb motor function and brain network function, defined as rsFC measured by BOLD signal analysis on fMRI, in patients with severe motor paralysis after stroke.

## Materials and Methods

### Study Design

This was a prospective case series study involving patients after stroke with hemiparesis.

### Participants

Eleven patients participated in this study (seven men and four women; five with right hemiparesis and six with left hemiparesis; age: 54.7 ± 10.8 years; height: 162.5 ± 7.7 cm; weight: 65.2 ± 13.7 kg; Table 1). All patients provided written informed consent to participate in this study, which was approved by the local ethics committee (Shonan Keiiku Hospital, No. 17-0005) and conformed to the Declaration of Helsinki. This study was registered as a clinical trial with the University Hospital Medical Information Network in Japan (UMIN Critical Trial Registry UMIN000032286). The inclusion criteria were: (a) diagnosis of unilateral stroke, not involving the cortex; (b) Stroke impairment assessment set (SIAS) of the finger function 1A (ability to flex the paretic fingers voluntarily but not to extend them; Sonoda et al., 1993; Chino et al., 1996); (c) passive range of motion greater than −30° for metacarpophalangeal joint extension; (d) time from stroke onset to be more than 4 months; (e) ability to walk independently in daily life with or without assistance; (f) age older than 18 years; and (g) not receiving other special rehabilitation or treatment for upper extremity paralysis such as transcranial magnetic stimulation, repetitive facilitative exercise, and botulinum toxin within 3 months.

**Table 1:** Clinical details of patients.

No.	Lesion side	Lesion location	TFO (months)	SIAS U/L (Proximal)
1	Left	Insula hemorrhage	31	1
2	Left	Corona radiata hemorrhage	44	3
3	Right	Putamen hemorrhage	41	3
4	Right	Subcortical hemorrhage	112	2
5	Left	Putamen hemorrhage	43	3
6	Left	Putamen hemorrhage	36	3
7	Right	Thalamus and corona radiata infarction	5	3
8	Left	Putamen hemorrhage	37	3
9	Right	Thalamus hemorrhage	67	3
10	Right	Putamen hemorrhage	9	2
11	Left	Thalamus hemorrhage	59	3

*TFO, Time from the onset; U/L, Upper Limb; SIAS, stroke impairment assessment set*.

The exclusion criteria were: (a) inability to understand the purpose and task of this study; (b) severe internal disorder of the heart and metabolism; and (c) less than 0 score (never become more than 1) on the questionnaire of body ownership or kinesthetic sensation (as described below) during the intervention period.

Edinburgh Handedness Inventory (Oldfield, 1971) was used to assess patients’ handedness before the intervention onset. All participants were right-handed (the scores were 84.0 ± 17.6%).

As a supplementary experiment, 30 healthy individuals (17 men and 13 women; age: 25.2 ± 4.8 years; height: 165.2 ± 8.6 cm; weight: 59.2 ± 12.3 kg) were included for the measurements of functional magnetic resonance imaging, in addition to the patients. They had no neurological or psychiatric diseases and all participants were right-handed as indicated through the Edinburgh Handedness Inventory test (healthy group scores 82.9 ± 21.0%). MRI data acquisition was performed only once during this study period, between 4:00 PM and 5:30 PM. They were instructed to perform normal daily activities on the day of the MRI scan.

### Interventions

**Figure 1:**
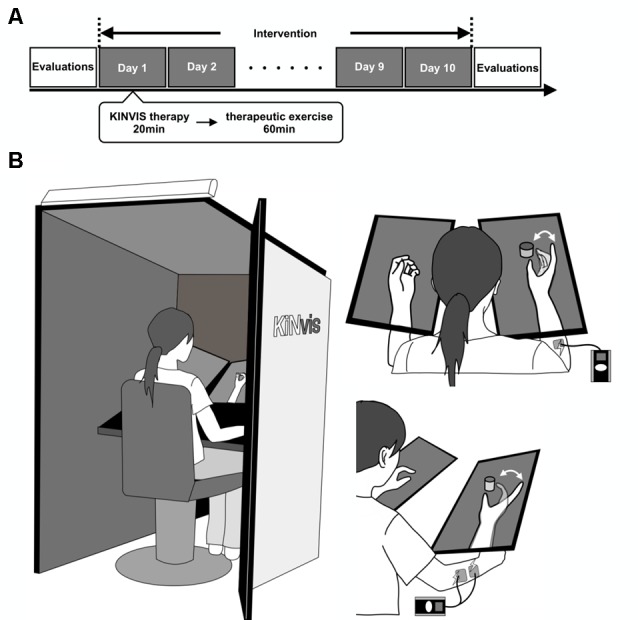
**(A)** Outline of the experimental plan. The experiment consisted of 10 days of intervention on weekdays and evaluations before and after the intervention. The intervention included conventional therapy for kinesthetic illusion induced by visual stimulation (KINVIS) therapy and therapeutic exercise (TherEX). KINVIS was applied for 20 min, and TherEX was applied after KINVIS therapy for 60 min by physical or occupational therapists. **(B)** KINVIS therapy set-up. KINVIS used the KiNvis™ System that consists of a table with two monitors, a chair for the patient to sit, both within a box to encourage concentration on the movie. Neuromuscular Electrical Stimulation was applied in combination with KINVIS. The patients’ arm was supported on a stand to avoid any somatosensory input.

The experiment consisted of 10 days of intervention on weekdays and evaluations before and after the intervention (Figure 1A). The intervention included KINVIS therapy and conventional therapeutic exercise (TherEX).

#### KINVIS

The cognitive phenomenon of KINVIS can be described as the feeling of one’s body moving during sensory input, even though the body is actually in a resting state.

The subjects were applied a visual stimulation for 20 min based on our previous report using the KiNvis™ system (Kaneko et al., 2016a). Furthermore, we applied neuromuscular electrical stimulation (NMES; NM-F1, Ito Company Limited) in combination with KINVIS (Figure 1B) as a KINVIS therapy. The patients were seated at a chair with their forearm on the table. The hand movement of the unaffected side was recorded before the intervention. The movement task involved hand opening and closing. This task was executed using the unaffected side and was flipped to mirror the movement of the affected side. Their arm was supported on a stand to avoid any somatosensory input.

Surface electrodes for stimulation were placed bilaterally on the skin overlying the extensor digitorum communis (EDC) muscle. Electrical stimulation was applied at an intensity higher than the motor threshold of the muscle (motor threshold, 1.0–1.2 times higher; frequency, 20 Hz; pulse width, 50 μs), while watching the movie during the finger-extension phase. During KINVIS therapy, the patients were relaxed and were instructed not to move their hands. KINVIS therapy was applied at two sets of 10 min, for a total of 20 min per day. Participants answered a question on body ownership and kinesthetic sensation in each set. Specifically, the question on body ownership was: “I felt the hand in the screen like own hand as if it was part of my own body during watching the movie”; this was based on a previous study on rubber hand illusion (Botvinick, 2004). The question on kinesthetic sensation was: “I had a feeling that my hand is actually moving during watching the movie” (Kaneko et al., 2015). Patients selected a level to agree/disagree from the following 7-point Likert scale for each question, as follows: −3, strongly disagree; −2, disagree; −1, somewhat disagree; 0, neither agree nor disagree; 1, somewhat agree; 2, agree; 3, strongly agree. The sense of ownership and kinesthetic sensation in each patient were calculated from the median score on the 7-point Likert scale in a period of 10 days.

#### TherEX

TherEX was applied for 60 min by a physical or occupational therapist after KINVIS therapy. Generally, the TherEX was composed of a kind of task-oriented exercise for finger movement and proximal joint movement, and stretching flexor muscles. For each patient, individual exercises for upper extremities were selected to gradually increase the difficulty level in a conventional way. Since suppression of the spasticity for each subject’s finger and wrist flexors were manually realized after KINVIS therapy, execution of some task had been possible according to individual condition. For example, a simple pinch and release a small object (from 5 mm to several centimeter balls, columns, etc.), which was size the subject could execute it with relaxed the proximal muscles and the wrist flexion position, was chosen as a task. We did not prescribe a special approach (i.e., repetitive transcranial magnetic stimulation, transcranial direct current stimulation, CIMT). Each patient was instructed to self-exercise, such as exercising and stretching their fingers, as well as performing activities of daily living assisted by a therapist.

### Outcome Measures

Clinical assessments of this study included measurements of upper limb motor function, spasticity, and actual use of the affected upper limb in activities of daily living. All clinical assessments were measured within an hour.

Fugl-Meyer assessment (FMA) was performed to assess upper extremity motor recovery. This test consisted of shoulder/elbow/forearm, wrist, hand movement and coordination. The FMA upper extremity motor score ranges from 0 to 66 (Platz et al., 2005). The Modified Ashworth Scale (MAS) was used to assess muscle tone in the 2nd to 5th finger flexor muscles, and wrist flexor muscles. The MAS is an ordinal scale with scores of 0, 1, 1+, 2, 3, 4 (Li et al., 2014). To calculate the mean value of MAS scores, score 1+ was transformed to 2, and scores 2, 3, and 4 were transformed to 3, 4, and 5.

The action research arm test (ARAT) was used to reflect motor function. The ARAT consist of four components; grasp, grip, pinch, and gross arm movement. This test involves transferring and picking up objects of different size. Each component’s score ranges from 0 to 4, and the total score ranges from 0 to 57 (Platz et al., 2005). The Box and Block Test (BBT) is one of the most common measures of motor function in stroke (Platz et al., 2005), and normative data are available for healthy adults (Mathiowetz et al., 1985). The score is calculated by the number of blocks transported in 60 s.

The motor activity log (MAL) assesses the actual use of upper limbs in daily living. This test is divided into the amount of use (AOU) and the quality of movement (QOM). The MAL is a self-report questionnaire inquiring about the amount and quality of daily use of the affected upper limb for 14 daily activities, such as holding a book and picking up a glass (Uswatte et al., 2005).

### MRI Data Acquisition

Two conditions of MRI data were obtained. First, resting-state fMRI was performed to measure rsFC. During this session, participants were instructed to keep their eyes open and maintain wakefulness, without moving their head. For the resting-state fMRI scanning session, a gradient-echo echo-planar sequence was used (3.75 × 3.75 × 3.5 voxels; echo time = 40 ms; repetition time = 2,500 ms, flip angle = 85°), in order to collect BOLD contrast data of 244 slices. Second, a T1-weighted MRI scan was acquired, and participants were asked to keep their eyes closed during this session. T1-weighted structural images were acquired as an anatomical reference (0.4688 × 0.4688 × 1.4 mm voxels; echo time = 3.076 ms; repetition time = 8.368 ms; flip angle = 12°). All MRI data were obtained using a 1.5-T MRI scanner with a head coil (Optima 450w, GE Healthcare, Chicago, IL, USA). MRI data acquisition was performed before and after the intervention for patients.

#### Resting-State fMRI Data Preprocessing

All data preprocessing sessions and analyses were carried out using CONN toolbox (Whitfield-Gabrieli and Nieto-Castanon, 2012) implemented on MATLAB (Mathworks Inc., Natick, MA, USA). CONN is a conjunction in Statistical Parametric Mapping 12 (SPM12; Well-come Department of Imaging Neuroscience, London, UK); therefore, the following preprocessing pipeline is SPM12 compliant. Image preprocessing consisted of: (1) reducing the signal for the first four slices for magnetization equilibrium effects and adaptation of the subjects to the circumstances; (2) flipping of all structural/functional data of right-hand affected patients using non-rigid reflection along the x-axis, in order to align the affected hemisphere on the left side; (3) functional realignment and unwarping; (4) slice-timing correction applied to the images; (5) creation of binary masks in gray matter, white matter, and cerebrospinal fluid images using structural segmentation; (6) denoising using outlier detection thresholded 97th percentile in normative samples of framewise displacement; (7) normalization to the EPI image template conforming to the Montreal Neurological Institute (MNI) space (2 mm iso voxels); (8) spatial smoothing with an isotropic Gaussian kernel of 7-mm full width at half maximum; and (9) band-pass filtering with a setting of 0.01–0.08 Hz.

#### ROIs in This Intervention

Twenty ROIs associated with sensory-motor function and KINVIS were defined as spherical seed regions with a radius of 6 mm. ROIs were defined in the motor-related activation area, KINVIS activated area, and supramarginal gyrus (SMG), based on previous fMRI studies (Mayka et al., 2006; Gentile et al., 2013; Kaneko et al., 2015). The names and MNI coordinates of ROIs are shown in Table 2. Each of 10 ROIs was created in the MNI space on the affected and unaffected hemispheres.

**Table 2:** Regions-of-interests associated with sensory-motor function and KINVIS.

Side	Anatomical labels of MNI atlas	Functional labels	MNI coordinates		
			*x*	*y*	*z*
Affected hemisphere	Precentral gyrus	PMd	−30	−4	58
	Precentral gyrus	PMv	−50	5	22
	Supplementary motor area	SMA	−2	−7	55
	Precentral gyrus	M1	−37	−21	58
	Postcentral gyrus	S1	−40	−24	50
	Inferior parietal lobule	IPL	−33	−45	51
	Superior parietal lobule	SPL	−36	−54	60
	Intra parietal sulcus	IPS	−51	−42	45
	Supramarginal gyrus	SMG	−52	−30	46
	Insula lobe	Insula	−35	20	5
Unaffected hemisphere	Precentral gyrus	PMd	30	−4	58
	Precentral gyrus	PMv	50	5	22
	Supplementary motor area	SMA	2	−7	55
	Precentral gyrus	M1	37	−21	58
	Postcentral gyrus	S1	40	−24	50
	Inferior parietal lobule	IPL	33	−45	51
	Superior parietal lobule	SPL	36	−54	60
	Intra parietal sulcus	IPS	51	−42	45
	Supramarginal gyrus	SMG	52	30	46
	Insula lobe	Insula	35	20	5

*List of regions-of-interest (ROIs) set based on the hypothesis. Anatomical labels are according to the Montreal Neurological Institute (MNI) atlas; the functional labels in each site and MNI coordinates of each hemispheric side of ROIs are shown. Abbreviations: PMd, dorsal premotor cortex; PMv, ventral premotor cortex; SMA, supplementary motor area; M1, primary motor cortex; S1, primary somatosensory cortex; IPL, inferior parietal lobule; SPL, superior parietal lobule; IPS, intra parietal sulcus; SMG, supramarginal gyrus*.

### Statistical Analyses

For motor function, a paired *t*-test was used to compare clinical assessments before and after the intervention. The statistical significance level was 5%. Statistical analyses were performed using SPSS, version 24.0 J (SPSS, Japan). Furthermore, we calculated the effect size of the clinical assessment scale using Cohen’s *d* statistics, and the magnitude of the difference between before and after the intervention was defined as small if *d* = 0.2, medium if *d* = 0.5, or large if *d* = 0.8, considering the clinical significance of the variables.

For rsFC scores, seed-based connectivity analysis was used to identify the brain regions temporally correlated using BOLD signal fluctuations in the ROIs. To represent the level of rsFC between each ROI and every location in the brain, this analysis used *Z*-scores which were the Fisher-transformed bivariate correlation coefficients between BOLD time-series averaged across all voxels within an ROI and an individual voxel.

In order to assess rsFC between an ROI and other regions at each measurement, we conducted one-sample *t*-test for *Z*-scores to search for ROI-to-Voxel with a particularly significant rsFC in each session (independently before and after the intervention), as reported by Zhang et al. (2016). Since Eklund et al. (2016) confirmed that the false positive rate is high when using the conventional threshold value, we searched for a site by the conservative threshold. As a result, threshold was set to the voxel level false-discovery-rate (FDR) corrected *p* < 0.01 and at cluster level FDR corrected *p* < 0.0001.

This study primarily focused on exploring whether the rsFC and motor functions would be indicated or whether the intervention in the patients with stroke would influence these relationships. Hence, *Z*-scores between significant clusters and ROIs were calculated to verify the correlation between *Z*-score and FMA upper extremity motor or ARAT total score. Similarly, for the stage of before intervention, the cluster mask of after intervention was used to calculate the *Z*-score between the same sites. Pearson’s product-moment correlation coefficient was used for this analysis. The statistical level was corrected to *p* < 0.025 using the Bonferroni method to avoid type 1 errors caused by verifying the correlation between ROI and motor function twice (i.e., FMA upper extremity motor and ARAT total score). In addition, if the correlation between *Z*-scores before or after the intervention and motor function was judged to be significant, testing the significance of the correlation coefficient was used to verify whether the correlation coefficient was significantly different. The significant threshold was corrected by the Bonferroni method to *p* < 0.05/*n* according to *n* times of tests.

## Results

KINVIS was induced in all patients. The 7-Likert scale [median (IQR)] scores on body ownership and kinesthetic sensation were 2 (2–2) and 2 (1–2), respectively. These results suggest that self-body cognitive effects during KINVIS were detected subjectively.

### Clinical Examination of Behavior

The results of clinical assessments are reported in Table 3. Regarding the FMA, the Shoulder/Elbow/Forearm score and, accordingly, the upper extremity motor score were significantly improved by the intervention (Shoulder/Elbow/Forearm: *p* = 0.003, effect size 0.63; upper extremity motor score: *p* = 0.003, effect size 0.65).

**Table 3:** Clinical examination.

	Before	After	*p*-value	Effect size *(d)*
Fugl-Meyer assessment scale
Shoulder/Elbow/Fore-arm	11.2 ± 2.9	13.4 ± 4.0**	0.003	0.63
Wrist	0.0 ± 0.0	0.1 ± 0.3	0.341	0.43
Hand	1.0 ± 0.0	1.0 ± 0.0	-	-
Upper extremity total score	12.2 ± 2.9	14.5 ± 4.0**	0.003	0.65
Modified Ashworth scale
2nd to 5th finger flexor muscles	3.7 ± 0.5	2.7 ± 1.0**	0.008	1.27
Wrist flexor muscles	3.0 ± 1.2	1.8 ± 1.1**	0.001	1.01
Action research arm test
Grasp	0.9 ± 1.5	1.6 ± 1.9	0.054	0.43
Grip	1.3 ± 1.4	1.9 ± 2.5	0.132	0.32
Pinch	0.2 ± 0.6	1.2 ± 2.1	0.085	0.64
Gross movement	2.9 ± 1.8	3.4 ± 1.9	0.138	0.25
Total score	5.3 ± 4.4	8.1 ± 7.6*	0.018	0.46
Box and Block test	0.5 ± 1.0	1.7 ± 3.0	0.066	0.57
Motor activity log				
Amount of use	0.2 ± 0.3	0.5 ± 0.5**	0.007	0.73
Quality of movement	0.3 ± 0.5	0.5 ± 0.5**	0.008	0.49

Paired *t*-test for significant differences compared to before the intervention; *p < 0.05 and **p < 0.01. Effect sizes were calculated using Cohen’s *d* statistic, and the magnitude of the difference after the intervention was defined as small if *d* = 0.2, medium if *d* = 0.5, or large if *d* = 0.8. For the FMA hand score, the *p*-value and effect size was not calculated because of the same value of average and standard deviation before and after the intervention.

The intervention significantly improved the MAS scores of 2nd to 5th finger flexor muscles and wrist flexor muscles (2nd to 5th finger flexor muscles: *p* = 0.008, effect size 1.28; wrist flexor muscles: *p* = 0.001, effect size 1.01), the total ARAT score (*p* = 0.018, effect size 0.46), and the AOU and QOM scores of the MAL (AOU: *p* = 0.007, effect size 0.73, QOM: *p* = 0.008, effect size 0.49), while the BBT score showed no significant improvement.

### Brain Functional Connectivity

**Figure 2:**
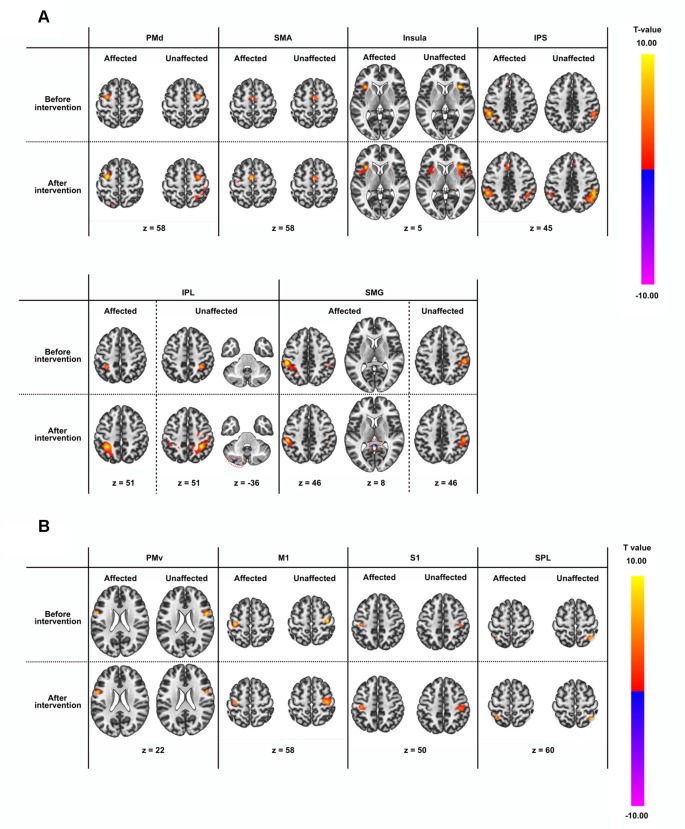
Results of seed-based functional connectivity in all regions-of-interest (ROIs). ROI names are defined in Table 2. Results before and after the intervention are displayed in rows, while those in the affected and unaffected hemispheres are displayed in columns. All connectivity maps were thresholded at voxel level false-discovery-rate (FDR) corrected at *p* < 0.01 and at cluster level FDR corrected at *p* < 0.0001. **(A)** ROIs showing significant intrahemispheric or interhemispheric resting-state brain functional connectivity (rsFC). **(B)** ROIs not showing significant intrahemispheric or interhemispheric rsFC apart from the rsFC to themselves.

Seed-based connectivity analysis of data acquired from 11 patients demonstrated several rsFC changes. Before the intervention period, there were several significant rsFC changes between an ROI in the affected and unaffected hemispheres, and other voxels (Table 4, Figure 2A). For interhemispheric rsFC, five ROIs in the parietal cortex of the affected hemisphere showed significant rsFC with areas of the contralateral homologous site. In contrast, after the intervention, significant and positive rsFCs with contralateral homologous sites were additionally represented in the inferior parietal lobule (IPL), intra-parietal sulcus (IPS), and insula (Table 4).

**Table 4:** Results list of resting-state functional connectivity.

			Before intervention			After intervention		
No.	ROI	Side	Clusters (*x y z*)	Voxels	Region of clusters	Clusters (*x y z*)	Voxels	Region of clusters
**(A)**								
1	Insula	Affected	56 12 2	32	Unaffected *FO* (PMv)	26 20 8	94	Unaffected *Insula* and FO (PMv)
2	Insula	Unaffected				−34 4 6	203	Affected *Insula* and FO (PMv)
3	IPS	Affected	52 −32 38	138	Unaffected *SMG*, AG and PO (IPL, IPS)	46 −46 52	548	Unaffected *SMG*, AG (IPL, IPS), SPL and LOC
4			−2 26 40	97	Affected *PaCiG* and SFG	−2 24 48	207	Affected/Unaffected *PaCiG* and SFG
5	IPS	Unaffected				−52 −46 48	246	Affected *SMG* and AG (IPL, IPS)
6	IPL	Unaffected				−30 −48 66	251	Affected SPL, *PostCG* (S1) and SMG
7						−30 −80 −36	110	Affected Cerebellum Crus 2*
8	SMG	Affected	48 −38 44	127	Unaffected *SMG*, SPL and AG (IPL, IPS)	52 −34 50	240	Unaffected *SMG* (IPL, IPS), and SPL
9						2 −40 8	45	Affected/Unaffected *Vermis 4 5* and PC*
**(B)**								
10	PMd	Affected	−16 −66 44	62	Affected LOC and *Precuneus* (SPL)	−18 −68 60	239	Affected LOC and *Precuneus* (SPL)
11						−42 −46 48	167	Affected *PostCG* (S1), SMG (IPL, IPS) and SPL
12	SMA	Affected				−42 −20 44	97	Affected preCG (M1), *PostCG* (S1) SMG (IPS)
**(C)**								
13	PMd	Unaffected				52 −28 50	595	Unaffected PreCG (M1), *PostCG* (S1), SMG and SPL
14	IPS	Unaffected				6 30 40	160	Affected/Unaffected *PaCiG* and SFG
15	IPL	Unaffected				22 −8 68	141	Unaffected *SFG*, PreCG and MidFG (PMd)

Voxel level threshold: false-discovery-rate corrected *p* < 0.01. Cluster level threshold: false-discovery-rate corrected *p* < 0.0001. Table of significant clusters associated with Figure 2 before and after the intervention in patients. The names of regions-of-interest (ROI) are defined in Table 2. ROI items that had no cluster except themselves were excluded. Clusters continuous from the ROI were removed from this table. The cluster-level threshold was false-discovery-rate (FDR) corrected at *p* < 0.0001. Table items **(A–C)** were created from connectivity patterns: (A) ROIs with interhemispheric connectivity before or after intervention; (B) ROIs with connectivity in the affected hemisphere before or after intervention; (C) ROIs with connectivity in the unaffected hemisphere before or after intervention. Header description: No., the number of ROI-to-clusters judged to be significant; Side, affected or unaffected hemisphere; Clusters, coordinates of cluster peak location in Montreal neurological Institute space; Voxels, cluster size; Region of clusters, brain regions of the cluster. The region corresponding to coordinates of cluster peak location are shown in italic. Abbreviations: FO, frontal operculum cortex; SFG, superior frontal gyrus; FP, frontal pole; SMG, supramarginal gyrus; AG, angular gyrus; PO, parietal operculum; SPL, superior parietal lobule; LOC, lateral occipital cortex; PaCiG, paracingulate gyrus; MidFG, middle frontal cortex; PreCG, precentral gyrus; PostCG, postcentral gyrus; PC, cingulate gyrus posterior division. *The clusters which negatively correlated with ROI.

There was a large number of significant intra- and inter-hemispheric rsFC in the healthy group. rsFC in patients after the intervention were common to rsFC represented in healthy group (Supplementary Figure S1, Table S1). The size of the region of the cluster was generally larger in the healthy group than the patients group. rsFC between IPL and SMG in the patient group did not contradict those in the healthy group.

### Correlation Between Brain Functional Connectivity and Motor Function

**Figure 3:**
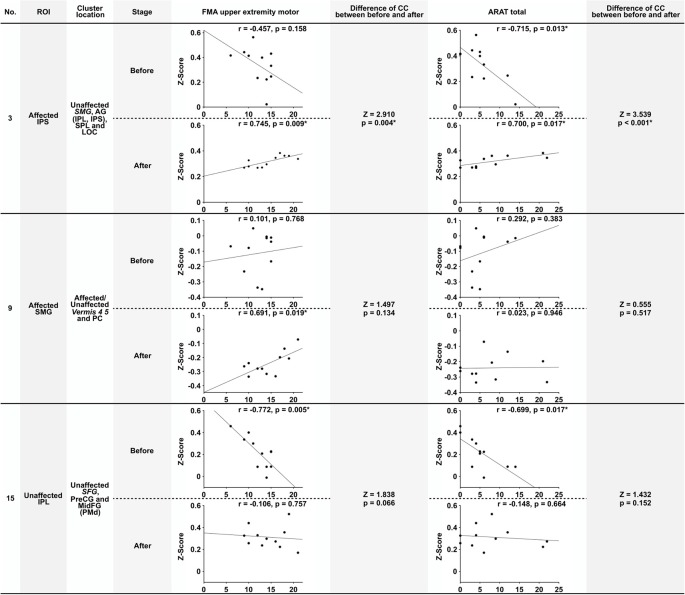
Scatter plots of significant correlation in Table 5, and the results of testing the significance of the correlation coefficient. This figure shows the scatter plots of the significant correlations shown in Table 5. The number (No), ROI name, and cluster location name of Columns are associated with Tables 4, 5. Stage means the assessment stage before and after the intervention. The correlation coefficient (*r*) and the probability value (*p*) shows above the scatter plot. The column of “Difference of CC (correlation coefficient) between before and after” shows the result of testing of the significance of the correlation coefficient (Z, *Z*-score; p, probability value). The significance probability defined *p* = 0.05/6 because a total of six tests were required. Values were marked *if they were below this threshold. Abbreviations: FMA upper extremity motor, the score of Fugl-Meyer assessment upper extremity motor function; ARAT total, Total score of Action research arm test; SMG, supramarginal gyrus; AG, angular gyrus; SPL, superior parietal lobule; MidFG, middle frontal cortex; PreCG, precentral gyrus; PC, cingulate gyrus posterior division.

The correlation between the *Z*-score and the motor functions was analyzed before and after the intervention and compared. The *Z*-score was calculated from the combination of significant ROI-to-cluster obtained from the results of the seed-based analysis. Table 5 indicates correlation coefficients and significances between functional connectivity from ROI-to-cluster and motor functions. In addition, Figure 3 shows the scatter plots of *Z*-score and the value of motor functions that demonstrated significant correlations among those shown in Table 5, and the results of examination for the significance of the correlation coefficients between stages of before and after the intervention.

**Table 5:** Correlation coefficient between resting-state functional connectivity and motor function.

				FMA upper extremity motor		ARAT total	
No.	ROI	Cluster location	Stage	*r*	*p*	*r*	*p*
1	Affected Insula	Unaffected *Insula* and FO (PMv)	Before	0.255	0.450	0.531	0.093
			After	0.600	0.051	0.298	0.373
2	Unaffected Insula	Affected *Insula* and FO (PMv)	Before	0.394	0.230	0.585	0.059
			After	0.133	0.698	0.578	0.063
3	Affected IPS	Unaffected *SMG*, AG (IPL, IPS), SPL and LOC	Before	−0.457	0.158	**−0.715**	0.013*
			After	**0.745**	0.009*	**0.700**	0.017*
4		Affected/Unaffected *PaCiG* and SFG	Before	−0.019	0.956	−0.257	0.446
			After	0.373	0.258	0.560	0.073
5	Unaffected IPS	Affected *SMG* and AG (IPL, IPS)	Before	−0.392	0.233	−0.586	0.058
			After	0.495	0.121	0.123	0.719
6	Unaffected IPL	Affected SPL, *PostCG* (S1) and SMG	Before	−0.138	0.685	−0.353	0.286
			After	0.191	0.574	0.231	0.494
7		Affected *Cerebellum Crus 2*	Before	0.266	0.428	0.360	0.277
			After	0.078	0.821	0.002	0.996
8	Affecrted SMG	Unaffected *SMG* (IPL, IPS) and SPL	Before	−0.002	0.996	−0.512	0.108
			After	−0.002	0.996	0.028	0.935
9		Affected/Unaffected *Vermis 4 5* and PC	Before	0.101	0.768	0.292	0.383
			After	**0.691**	0.019*	0.023	0.946
10	Affected PMd	Affected LOC and *Precuneus* (SPL)	Before	−0.273	0.417	−0.267	0.428
			After	0.412	0.208	0.060	0.860
11		Affected *PostCG* (S1), SMG (IPL, IPS) and SPL	Before	−0.110	0.747	−0.594	0.054
			After	0.369	0.264	0.379	0.250
12	Affected SMA	Affected preCG (M1), *PostCG* (S1) and SMG (IPS)	Before	0.205	0.545	−0.181	0.594
			After	−0.218	0.519	0.203	0.549
13	Unaffected PMd	Unaffected PreCG (M1), *PostCG* (S1), SMG and SPL	Before	−0.507	0.111	−0.565	0.070
			After	−0.216	0.523	0.008	0.982
14	Unaffected IPS	Affected/Unaffected *PaCiG* and SFG	Before	−0.207	0.541	−0.130	0.704
			After	−0.272	0.419	−0.540	0.086
15	Unaffected IPL	Unaffected *SFG*, PreCG and MidFG (PMd)	Before	**−0.772**	0.005*	**−0.699**	0.017*
			After	−0.106	0.757	−0.148	0.664

Significant correlation coefficient; *p < 0.025. Correlation coefficient and significance between functional connectivity from ROI-to-cluster and motor function. These numbers in the first column are associated with Table 4 and Figure 3. For each of ROI, the results of correlation coefficient (r) and probability value (p) between Z-score and motor function at the stage of before or after intervention are described. This table shows the correlation between the Z-score of ROI-to-cluster calculated from functional connectivity results after the intervention and motor function. The threshold of significant correlation coefficient is *p* = 0.025, and a value represented in bold and marked *indicates significance. ROI No. 3: connectivity between the bilateral intraparietal sulcus was positively correlated between the FMA upper extremity motor and ARAT total after the intervention. Before the intervention, this connectivity was negatively correlated with ARAT total; ROI No. 9: connectivity between supramarginal gyrus in affected hemisphere only and affected or unaffected cerebellum vermis showed positive correlation only after the intervention; ROI No. 15: connectivity between inferior parietal lobule and premotor cortex was only positively correlated before the intervention.

Before the intervention, the connectivity of the bilateral parietal cortex was negatively correlated with ARAT total (Table 5, No. 3). Furthermore, the connectivity of IPL and PMd was also negatively correlated with the motor functions before the intervention (Table 5, No. 15). Connectivity between the bilateral IPS (including IPL, SPL, and LOC in the unaffected hemisphere) was positively correlated between the FMA upper extremity motor and ARAT total after the intervention. Moreover, connectivity between SMG in the affected hemisphere only and the affected or unaffected cerebellum vermis showed a positive correlation only after the intervention (Table 5, No. 9), although this correlation was not significant. Among the significant correlations in each stage, the connectivity that showed the significant difference before and after the intervention was that of the bilateral IPS (FMA upper extremity motor: *Z* = 2.910, *p* = 0.004; ARAT total: *Z* = 3.539, *p* < 0.001). Therefore, significant changes in the relationship between the movement functions and rsFC in the bilateral IPS after the intervention were demonstrated.

## Discussion

The present research was the first prospective study demonstrating the effect of KINVIS therapy with TherEX on motor function and brain function in patients with stroke. As we expected from our preliminary study (Kaneko et al., 2016a), the motor functions examined with FMA and MAS improved after the therapeutic intervention period. The improvements in MAS reached a minimum of clinically important differences (Platz et al., 2005; Li et al., 2014). Furthermore, as the main result for rsFC, a significant change in the relationship between the motor functions and rsFC of bilateral IPS occurred parallel to the motor functional improvement. Although significant rsFC changes between an ROI and the clusters in each stage were also detected, they did not indicate direct comparison between stages. Clinically, the meaning of the differences in the detected significant pairs of the ROIs and the clusters in each stage was not indicted. The number of ROIs with significant rsFCs was limited before the intervention; however, 15 common pairs (Table 4) of functionally connected ROIs were detected after the intervention. Therefore, we suggest that these pairs indicate significance in each stage. However, a future study including a control group should be designed with a direct comparison between stages.

The intervention adopted in the present study was “KINVIS,” which is a novel approach to hemiparesis developed by our group. From the results for the subjective score on the embodiment, body ownership, and KINVIS, this novel approach may influence the brain system associated with embodiment. We reported a number of fundamental studies regarding the physiological effect of this approach and the suspected clinical effects on paralytic extremities (Kaneko et al., 2007, 2015, 2016a,b; Aoyama et al., 2012). This technology can be viewed as a kind of virtual reality using embodied-visual feedback; however, it is not just virtual reality but can also induce embodied cognitive changes in one’s body and physiological effects on motor-associated areas in the brain. The cognitive effects induced during KINVIS therapy include body-ownership of the artificial body in the movie, kinesthetic perception, and/or a kind of desire or will to move one’s body. The point of this technology was to induce the illusory kinesthetic perception of the subject’s body, in contrast to the control condition in the previous experiment that involved a simple movement observation without such perception. Since our previous experiments indicated the physiological advantages of KINVIS as a therapeutic intervention for hemiparesis after stroke (Kaneko et al., 2007, 2015, 2016a,c; Aoyama et al., 2012), we conducted this first clinical trial.

An important feature of this study was that the participants were patients with severe hemiparesis and in the chronic phase after stroke. There are not many studies aimed at improving motor function in patients with chronic severe hemiparesis, as it is extremely difficult to apply a TherEX for hand and finger movement function. To compare the effect of various interventions to that of KINVIS, we searched PubMed for articles including the following key terms: stroke, chronic, upper limb, severe, and rehabilitation. As a result, we extracted 143 articles. Articles before 2014 were drawn from the Cochrane review (Pollock et al., 2014). There were 20 articles in which FMA or MAS were evaluated, and the effect size could be calculated. Since the patients in the present study were relatively severely paralyzed, articles in which the participants’ FMA score was less than 15 points were selected to discuss the effect size of interventions. Using these inclusion criteria, six articles were selected (Stinear et al., 2008; Ramos-Murguialday et al., 2013, 2019; Colomer et al., 2016; Grimm et al., 2016; Carda et al., 2017). Among those, the effect size was more than medium (Cohen’s *d* > 0.5) in only two articles. The effect size in our study was comparable to that of the previous six articles, indicating that the KINVIS therapy may be a powerful, effective approach for chronic, severe paralysis after stroke, and further clinical research is required. Importantly, the duration of intervention in our study was much shorter than that in the previous six studies. The duration of the intervention was at least 2 weeks in one study and more than 3 weeks in the other five studies. The intervention duration in our study was only 10 days, which is an advantage of our approach. The effect of long-term KINVIS therapy is not clear and should be assessed in future studies.

Our study also revealed a significant effect of the intervention on the spasticity of flexor muscles in the upper extremity, and the difference of MAS score reached the Minimal Clinically Important Difference (Platz et al., 2005; Li et al., 2014). As no previous study has indicated an effect on spasticity except for medication studies (Francis et al., 2004; Chen et al., 2005; Barros Galvão et al., 2014), this result is of great clinical importance. Moreover, the decrement in spasticity in the finger and wrist joint flexor muscles would now allow patients to proceed to TherEX, particularly in the case of patients with severely paralyzed upper extremities, who are in a sustained clenching state, making it impossible to apply any TherEX without KINVIS therapy. This decrement in spasticity suggests that KINVIS therapy is better applied before TherEX. There is currently no study reporting similar results, thus this new approach may be useful in improving paralysis therapy. One limitation of this study is the lack of control data; however, these positive changes in motor functions after the novel intervention strategy provide significant insight for future clinical studies.

As a noteworthy result indicated in the present study, a strong correlation between rsFC in the bilateral parietal cortex and the motor functions after the intervention period was demonstrated. This result possibly suggests that rsFC in the bilateral IPS in a severely paralyzed patient with stroke in the chronic phase may be a prognostic indicator for motor function recovery potential; controlled clinical trials may clarify this important matter in the near future. There was a negative correlation between rsFC of the bilateral IPS and motor function of the ARAT before the intervention period, and the change in the correlation towards the positive direction after the intervention. We estimate that the positive correlation observed after the intervention period may represent the capability a patient essentially possessed, which is either real or masked. Since the negative correlation between rsFC and motor function may be contaminated by the brain functional disruption owing to the stroke and/or negative adaptation to dis-use of the upper limb owing to sensory-motor functional paresis, we speculate the present intervention restored and advanced the sensory-motor and brain function in parallel. A similar phenomenon was demonstrated in a previous study from another group (Marumoto et al., 2013), and it may represent a common state in patients with stroke. The mechanism underlying these relationships is an interesting discussion point and it should be investigated in a future study. Also, connectivity between SMG in the affected hemisphere and the affected or unaffected cerebellum vermis showed a positive correlation with FMA only after the intervention. Furthermore, connectivity between the IPL and PMd in the unaffected hemisphere was also negatively correlated before the intervention. It would be clinically meaningful if the disappearance of this negative correlation in the unaffected hemisphere reflects the abnormal compensation of brain hyperactivity, as indicated in previous studies (Nowak et al., 2008; Dodd et al., 2017). If our novel approach can reduce contralesional hyperactivity, this would indicate its clinical potential and relevance. Besides, significantly increased connectivity was demonstrated in PMd with the parietal cortex in the affected hemisphere in both hemispheres after the intervention period. The anatomical fibers connecting the parietal cortex and PMd can be interpreted as the association fiber network (the superior longitudinal fasciculus fiber tracts) that includes the network predominantly involved when humans recognize changes in their position (Amemiya and Naito, 2016; Naito et al., 2016). The brain networks that were demonstrated to change in this study should be discussed, and whether these are associated with the reconstruction of body-schema alterations performed by KINVIS should be investigated in future studies. On the other hand, among these significant correlations, the connectivity that showed a significant difference between the stages before and after the intervention was that of the bilateral IPS only. We estimate that the rsFC bilaterally around the IPS may be key in connectivity, and is possibly associated with the functional improvement in the subject group recruited in this study. The pairs of brain areas where changes were indicated after simple TherEX in previous reports were homologous ROIs constituting the sensory-motor network (Carter et al., 2010; Urbin et al., 2014) and M1 (Carter et al., 2010, 2012; Wang et al., 2010; Westlake and Nagarajan, 2011; Urbin et al., 2014; Fan et al., 2015) only. Our results indicated homologous ROIs were correlated in the insula and areas of the parietal cortex (IPS, IPL, and SMG). Changes in these networks have not been previously reported, especially the change in the relationship between motor function and rsFC, which may be of clinical relevance.

Common brain areas between this and our previous study (Kaneko et al., 2015) that were associated with the effect of the KINVIS intervention were the SMA, PMd, SPL, IPL, and insula. These ROIs indicated a stronger intra-hemispheric correlation. We also found that the pairs of ROIs in the rostral-caudal direction, which may be connected *via* association fibers, were more strongly correlated after than before the intervention. The actual functional importance of this finding is not clear. However, the present results suggest that changes in intra-hemispheric rsFC in the uninvolved brain areas may be important for the recovery of motor function and spasticity. This neural plasticity was induced within only 10 days in patients in the chronic phase after stroke and occurred in parallel with the behavioral improvement. Our future objective is to decipher the network associated with this behavioral improvement.

Age-matched control trials are still needed in the future to gain further insight into the mechanisms involved in this phenomenon and to identify the brain networks that should be targeted in therapeutic approaches for the recovery of motor function. However, the functional improvement of the motor function and the indicated difference in the relationship between rsFC and motor function after combination approach of KINVIS therapy and TherEX in a severely paralyzed chronic phase patient with stroke were both clinically relevant and novel.

## Conclusions

This prospective exploratory clinical trial provided the first evidence that a combination therapeutic approach, i.e., KINVIS therapy with TherEX, may improve the motor functions in patients with severe hemiparesis in the chronic phase after stroke, as predicted by our preliminary report (Kaneko et al., 2016b). Our results are comparable to those previously reported in studies including patients who were not as severely paralyzed as in our trial.

Furthermore, changes of the relationship (negative to positive) between the motor functions and the interhemispheric rsFC of the bilateral IPS, and disappearance of the significant negative correlation between the motor functions and the intra-hemispheric rsFC of IPL in the unaffected side and PMd were parallelly demonstrated with behavioral changes. rsFC for the interhemispheric IPS and intrahemispheric IPL and PMd may be a possible regulatory factor for improving motor function and spasticity.

## Data Availability Statement

All datasets generated for this study are included in the article/Supplementary Material.

## Ethics Statement

The studies involving human participants were reviewed and approved by Shonan Keiiku Hospital Ethics Committee, 4360, Endo, Fujisawa, Kanagawa, Japan. The patients/participants provided their written informed consent to participate in this study.

## Author Contributions

KS, KA, and ML recruited patients and medically charged patients’ conditions. FK managed the quality of clinical interventions and examinations. MY and MO collected the clinical examinations data and analyzed data of clinical examination and fMRI data. FK and KS conceived and designed the study. FK and partially all other authors wrote the article. KS, KA, and ML critically reviewed the manuscript.

## Conflict of Interest

ML and FK are the founding scientists of Connect Inc., a commercial company for the development of rehabilitation devices since May 2018 (ML) and October 2018 (FK). This company does not have any relationship with the device or setup used in the current study. The remaining authors declare that the research was conducted in the absence of any commercial or financial relationships that could be construed as a potential conflict of interest.

## Abbreviations

KINVIS: Kinesthetic perceptional illusion by visual stimulation
TherEX: Therapeutic exercise
rsFC: Resting-state brain functional connectivity
fMRI: Functional magnetic resonance imaging
CIMT: Constraint-induced movement therapy
M1: Primary motor
S1: Somatosensory
BOLD: Blood-oxygen-level-dependent
ROIs: Regions of interest
TFO: Time from onset
U/L: Upper Limb
SIAS: Stroke impairment assessment set
NMES: Neuromuscular electrical stimulation
EDC: Extensor digitorum communis
FMA: Fugl-Meyer assessment
MAS: Modified ashworth scale
ARAT: Action research arm test
BBT: Box and Block Test
MAL: Motor activity log
AOU: Amount of use
QOM: Quality of movement
MNI: Montreal Neurological Institute
SMG: Supramarginal gyrus
FDR: False discovery-rate
IPL: Inferior parietal lobule
IPS: Intra parietal sulcus
LOC: Lateral occipital cortex
PMd: Dorsal premotor cortex
SMA: Supplementary motor area
SFG: Superior frontal gyrus
SPL: Superior parietal lobule.
