# Supplementary material for: A Case Series Clinical Trial of a Novel Approach Using Augmented Reality That Inspires Self-body Cognition in Patients With Stroke: Effects on Motor Function and Resting-State Brain Functional Connectivity
Source: Front Syst Neurosci. 2019 Dec 17;13:76. doi: 10.3389/fnsys.2019.00076 (PMC6929676; doi:10.3389/fnsys.2019.00076)
Supplement: Supplementary file 1 [file Data_Sheet_1.pdf]

Supplementary Material

Supplementary figure S1. Results of seed-based functional connectivity in all regions-of-interest (ROIs) in the healthy group

The resting-state brain functional connectivity (rsFC) in the healthy group is demonstrated in this Supplementary Figure S1. The notations are the same as the result of patients group in Figure 2. Therefore, 6 ROIs showing significant intrahemispheric or interhemispheric rsFC are in A, and 4 ROIs not showing significant intrahemispheric or interhemispheric rsFC apart from the rsFC to themselves are in B. The threshold of all connectivity maps was determined at voxel level false-discovery-rate (FDR) corrected at $p < 0.01$ and at cluster level FDR corrected at $p < 0.0001$.

Abbreviations: PMd, dorsal premotor cortex; PMv, ventral premotor cortex; SMA, supplementary motor area; M1, primary motor cortex; S1, primary somatosensory cortex; IPL, inferior parietal lobule; SPL, superior parietal lobule; IPS, intra parietal sulcus; SMG, supramarginal gyrus.

Supplementary table S1. Results of resting-state brain functional connectivity in the healthy group

(A)

No.	ROI	Side	Clusters (x y z)			Voxels	Region of clusters
1	Insula	Left	-36	20	4	32184	Left/Right *Insula*, AC, SFG (PMd) and FO (PMv)
2	Insula	Right	36	20	4	36437	Left/Right *Insula*, FP, AC, SFG (PMd) and FO (PMv)
3	IPS	Left	50	-40	46	4013	Right *SMG*, AG (IPL, IPS), SPL, LOC and Precuneus
4			-26	4	50	2127	Left/Right SFG and PaCiG<br>Left *MidFG* and PreCG (PMd)
5	IPS	Right	-56	-48	40	3335	Left *SMG*, AG, PO (IPL, IPS), SPL and LOC
6	IPL	Right	-42	-42	52	4519	Left PostCG (S1), *SMG* (IPL, IPS), SPL, LOC and Precuneus
7			-4	-44	-12	2521	Left/Right PC, Precuneus, Vermis 4 5 and Cerebellum 3*
8	SMG	Left	42	-38	56	4441	Right PostCG (S1), AG, *SMG* (IPL, IPS), SPL and LOC
9			-4	-44	2	2521	Left/Right PC, Precuneus, *Vermis 4 5* and Cerebellum 3 4 5*

*: The clusters which negatively correlated with ROI

(B)

No.	ROI	Side	Clusters (x y z)			Voxels	Region of clusters
10<br>11	PMd	Left	-30	-4	60	10226	Left/Right *SFG* (PMd), PreCG (M1), SMA, Precuneus (SPL)<br>Left PostCG (S1), SMG, LOC and SPL
12	SMA	Left	-2	-6	56	31485	Left/Right *SMA*, PreCG (M1), PostCG (S1), CO and AG (IPL, IPS)

(C)

No.	ROI	Side	Clusters (x y z)			Voxels	Region of clusters
13	PMd	Right	30	-4	58	17224	Left/Right SPL, SMGLOC, *SFG* (PMd), PreCG (M1), PostCG (S1), Precuneus, SMA, AC and PO (IPS, IPL)
14	IPS	Right	30	22	-2	12880	Right PaCiG FP, *IFG* (PMv), MidFG, SFG (PMd) and PreCG (M1)
15	IPL	Right	28	0	64	2419	Right PreCG (M1), IFG (PMd), MidFG, *SFG* (PMd) and SMA

Voxel level threshold: false-discovery-rate corrected $p < 0.01$

Cluster level threshold: false-discovery-rate corrected $p < 0.0001$

Table of significant clusters associated with the healthy group.

In this supplementary table, clusters in the healthy group corresponding to the results of patients obtained before and after the intervention are described. The names of regions-of-interest (ROI) are defined in Table 2. ROI items that had no cluster except themselves were excluded. Clusters continuous from the ROI were removed from this table. The cluster-level threshold was false-discovery-rate (FDR) corrected at $p < 0.0001$. Table items (A), (B), and (C) were created from connectivity patterns in the results of patients in Figure 2: (A) ROIs with interhemispheric connectivity before or after the intervention; (B) ROIs with connectivity in the affected hemisphere before or after the intervention; (C) ROIs with connectivity in the unaffected hemisphere before or after the intervention.

Header description: No., the number of ROI-to-clusters judged to be significant; Side, left or right hemisphere; Clusters, coordinates of cluster peak location in Montreal neurological Institute space; Voxels, cluster size; Region of clusters, brain regions of the cluster. The region corresponding to the coordinates of cluster peak location are shown in italics.

Abbreviations: FO, frontal operculum cortex; AC, cingulate gyrus anterior division; SFG, superior frontal gyrus; FP, frontal pole; SMG, supramarginal gyrus; AG, angular gyrus; PO, parietal operculum; SPL, superior parietal lobule; LOC, lateral occipital cortex; PaCiG, paracingulate gyrus; MidFG, middle frontal cortex; PreCG, precentral gyrus; PostCG, postcentral gyrus; PC, cingulate gyrus posterior division; CO, central opercular cortex IFG, inferior frontal gyrus.

frontiers
