# Supplementary figures and images for: A Case Series Clinical Trial of a Novel Approach Using Augmented Reality That Inspires Self-body Cognition in Patients With Stroke: Effects on Motor Function and Resting-State Brain Functional Connectivity
Source: Front Syst Neurosci. 2019 Dec 17;13:76. doi: 10.3389/fnsys.2019.00076 (PMC6929676; doi:10.3389/fnsys.2019.00076)

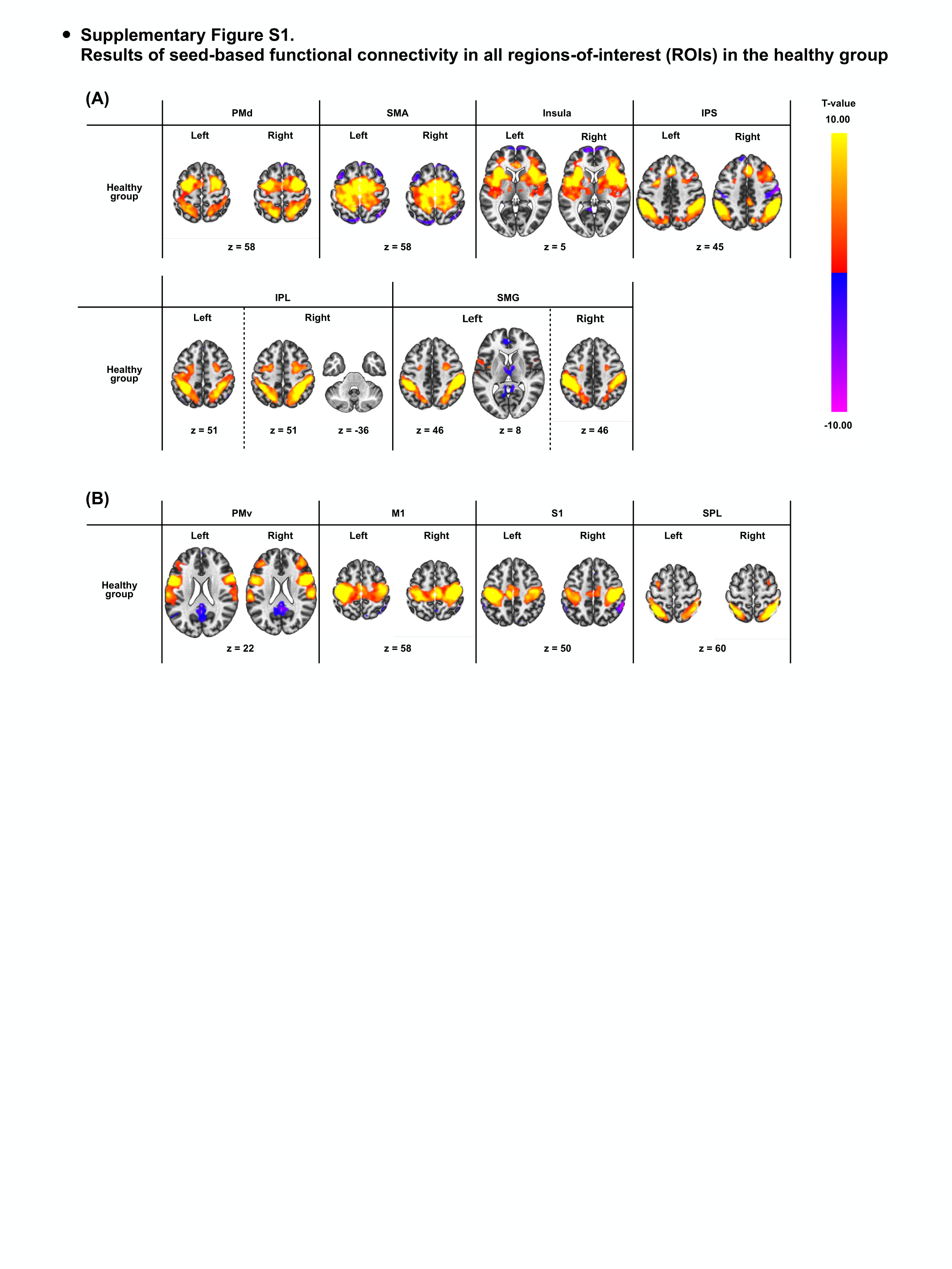

Supplement: Supplementary file 2 [file Image_1.tiff]
